# The DMCdrive: practical 3D-printable micro-drive system for reliable chronic multi-tetrode recording and optogenetic application in freely behaving rodents

**DOI:** 10.1038/s41598-020-68783-9

**Published:** 2020-07-16

**Authors:** Hoseok Kim, Hans Sperup Brünner, Marie Carlén

**Affiliations:** 1 Department of Neuroscience, Karolinska Institutet, 171 65 Stockholm, Sweden; 2 Department of Biosciences and Nutrition, Karolinska Institutet, 141 83 Huddinge, Sweden

**Keywords:** Neuroscience, Neuronal physiology

## Abstract

Electrophysiological recording and optogenetic control of neuronal activity in behaving animals have been integral to the elucidation of how neurons and circuits modulate network activity in the encoding and causation of behavior. However, most current electrophysiological methods require substantial economical investments and prior expertise. Further, the inclusion of optogenetics with electrophysiological recordings in freely moving animals adds complexity to the experimental design. Expansion of the technological repertoire across laboratories, research institutes, and countries, demands open access to high-quality devices that can be built with little prior expertise from easily accessible parts of low cost. We here present an affordable, truly easy-to-assemble micro-drive for electrophysiology in combination with optogenetics in freely moving rodents. The DMCdrive is particularly suited for reliable recordings of neurons and network activities over the course of weeks, and simplify optical tagging and manipulation of neurons in the recorded brain region. The highly functional and practical drive design has been optimized for accurate tetrode movement in brain tissue, and remarkably reduced build time. We provide a complete overview of the drive design, its assembly and use, and proof-of-principle demonstration of recordings paired with cell-type-specific optogenetic manipulations in the prefrontal cortex (PFC) of freely moving transgenic mice and rats.

## Introduction

Tetrode-containing micro-drives have long been an important technique for recordings of extracellular neuronal signals in behaving mice^1^ and rats^2^. More recently developed techniques, such as neuronal population calcium imaging^3^ and high-density silicon probes^4^ are superior to micro-drive arrays in respect to the number of neurons that can be recorded simultaneously. Despite this, there is still a range of application areas where micro-drive arrays are the method of choice, particularly for chronic recordings of action potential and local field potential (LFP) activity in freely moving rodents, including in conjunction with optogenetics. We here present a low-cost, easy to assemble micro-drive for tetrode recordings in conjunction with optogenetics (the DMCdrive). The strategy for the design has been to construct a user-friendly low size-low weight drive that reliably provides a) single-unit recordings over time b) persistent precise and predictable movement of all tetrodes c) efficient opto-tagging and optical manipulation of neurons. The drive consists of a few lab-made parts, printed using a standard 3D printer, a custom-made electronic interface board (EIB), and parts available off-the-shelf, and the easy-to-assemble design makes the drive particularly suitable for researchers with no or little experience of drive building. The production cost of the 3D printed material is ~ 1$, and the total cost for one drive ~ 40$ (six 3D-printed parts, an EIB with an 18-pin Omnetics connector, eight screws and a screw nut).

The DMCdrive holds four tetrodes, and is designed for movement (in depth) of the tetrodes at any time, an approach holding several advantages. First, as the neuronal signal deteriorates over time (likely due to the formation of a glial sheath that shields the electrodes from active neurons^5,6^), moving of the electrodes will in many cases restore the signal and provide stability of the recording conditions^6,7^. Second, adjustment of the depth of recording electrodes enables the recording of a new set of neurons, increasing the total number of neurons recorded per animal. However, if the moving force fails to move the electrodes through the glial sheath, the neural signal cannot be restored, and new neurons cannot be recorded. For this reason, reliable movement of all tetrodes was of high priority in the design of the drive, and by moving all tetrodes together the DMCdrive optimizes repositioning of the recording sites. Further, for precise targeting of recordings to specific locations, and estimation of the recording location after moving of the tetrodes, accurate depth movement of the tetrodes is of high importance. To achieve this, the movement of the tetrodes in the DMCdrive is controlled by a single, easily accessible shuttle screw, reliably moving the tetrodes 350 μm/360°. However, for the recording of neurons in e.g. curved brain structures such as the hippocampal formation drive designs allowing adjustment of individual tetrodes might be preferable.

Optogenetics in conjunction with extracellular recordings is a powerful method to study neural circuit function^8^. Optical tagging of neurons expressing light-activated opsins enables identification of the activity of select neurons, e.g., of specific neuron types^9,10^ or specific projections^11^. In addition, the inclusion of optogenetics allows for the manipulation of a neuronal population and recording of the effects on single-unit and/or LFP activities in the local network^12^. The DMCdrive is designed for inclusion of a static optical fiber, and integrates neurophysiological recordings with highly reliable manipulation of light-sensitive neurons. We provide a complete overview of the design of the drive, its assembly and use. For a demonstration of the utility of the drive, data from long-term (35 days post-implantation) and stable recordings in the PFC of freely moving transgenic mice and rats is provided, and includes opto-tagging of prefrontal inhibitory interneurons expressing parvalbumin (PV) and optogenetic entrainment of prefrontal gamma oscillations.

## Results

### Fabrication and assembly of the DMCdrive

**Figure 1:**
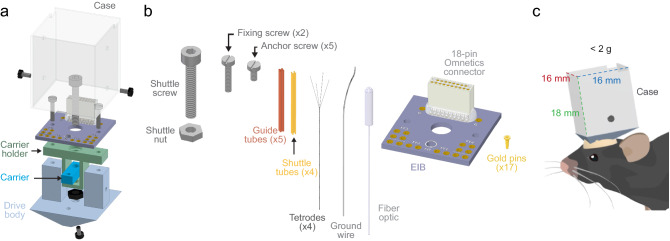
Schematic of the DMCdrive and component parts. (**a**) Isometric illustration of the functional parts of the DMCdrive. Four of the six 3D-printed parts are indicated (case, carrier holder, carrier, and drive body, respectively). (**b**) Parts needed, in addition to the 3D-printed parts, to build a DMCdrive for multi-tetrode recordings in conjunction with optogenetic manipulations. (**c**) Illustration of a mouse implanted with a DMCdrive. Outside measurements of the case: height; 18 mm (green dotted line), width and length; 16 mm (red and blue dotted line, respectively). Assembled with four tetrodes, an EIB, a ground wire and an optical fiber the drive weighs approximately 2 g.

**Figure 2:**
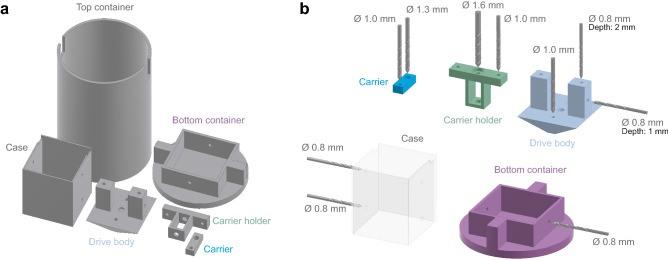
3D printing. (**a**) All 3D-printed parts of the DMCdrive, in a layout suitable for printing. The parts can be printed in less than two hours using a normal desktop 3D printer (Makerbot Replicator, Makerbot). (**b**) To ensure the correct sizing of screw holes, drilling of the holes is recommended after printing.

The DMCdrive consists of 3D-printed parts, commercially available parts, and a custom-made EIB designed to support 16 channels/4 tetrodes and one optical fiber (Fig. 1a, b). All required design files and a list of the materials, small parts, and tools needed for building of the drive are freely available at https://carlenlab.org/data and https://github.com/kimhos/DMCdrive. The focus of the drive design has been on functional fidelity, but also on very fast assembly, and minimization of the size and weight (16 × 16 × 18 mm, width x length x height; weight < 2 g; Fig. 1c). 3D-printing, drive assembly, tetrode and fiber optic loading, and testing of the impedance of the tetrodes can be achieved in 4 h with some practice. The six 3D-printed parts, designed using a 3D CAD modeling software (Autodesk Inventor, Autodesk), can be printed using a standard, low-cost 3D printer (FFF type, fused filament fabrication) in less than 2 h (Fig. 2a). The 3D-printed parts are printed together in one structure and should be cleaned from residual debris and structure (e.g., using an art knife). Screw holes in the printed drive parts should be carefully cleaned by drilling with appropriately sized drill bits (Fig. 2b).

**Figure 3:**
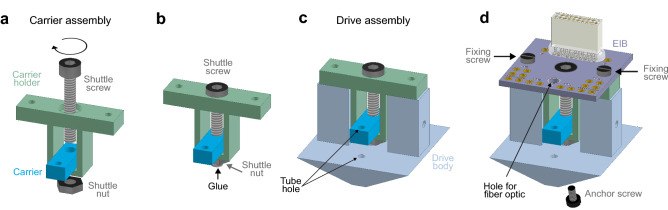
Drive assembly. (**a**) Insert the shuttle screw (M1.6 × 10 mm, hex socket head) into the carrier holder to connect the carrier with the carrier holder (‘carrier assembly’). Install the shuttle nut (M1.6, hex nut) on the shuttle screw. (**b**) Secure the shuttle nut to the shuttle screw with glue (black arrow). (**c**) Place the carrier assembly in the drive body (‘drive assembly’). (**d**) Connect an EIB holding an 18-pin Omnetics connector (A79042-001, Omnetics) to the drive assembly with two fixing screws (M1 × 5 mm thread length). Add 1–3 anchor screws (M1 × 2 mm thread length) to the bottom of the drive body for later securing of the drive to the dental cement surrounding the craniotomy.

**Figure 4:**
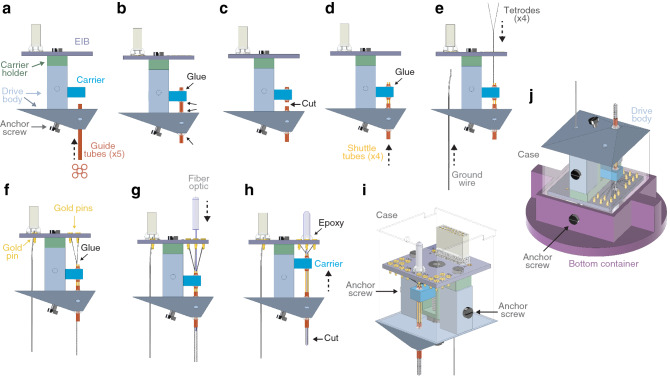
Tetrode and fiber optic loading. (**a**) Side view of the DMCdrive. Start by moving the carrier to the bottom of the drive. Insert the five guide tubes (0.0120″/0.0140″, ID/OD) into the drive body. The configuration of the guide tubes is dictated by the desired arrangement of the tetrodes and the optical fiber (e.g., round or flat). (**b**) Glue (arrows) the guide tubes to the drive body and carrier. (**c**) Cut (arrow) the guide tubes between the drive body and the carrier. (**d**) Insert four shuttle tubes (0.0049″/0.0064″, ID/OD) into four of the guide tubes. The fifth guide tube will hold the optical fiber. Glue (arrow) the shuttle tubes to its respective guide tube. (**e**) Insert the ground wire, without insulation at the ends, into and through the drive body from below. Place one tetrode in each shuttle tube. (**f**) Secure the ground wire and the tetrodes to the EIB using gold pins. To enable movement of the tetrodes, glue (arrow) the tetrodes to its respective shuttle tube. (**g**) Insert the fiber optic (flat tip fiber: multimode, 200 µm core, 0.22–0.5 NA; ferrule: Ø1.25 mm, 6.4 mm long, Ø230 µm bore; Thorlabs) through the EIB into the empty guide tube. (**h**) Glue (arrow) the fiber optic to the EIB. Move the carrier up as far as possible. (**i**) Place the assembled drive in the case, and secure the drive to the case using two anchor screws (‘finished drive’). (**j**) For storage and transport of the drive, place the finished drive upside down in the bottom container and secure with anchor screws, and then place the top container over the drive. *ID/OD* inner/outer diameter.

The drive assembling starts by building of the carrier assembly, which includes securing of the tetrode carrier to the carrier holder using a shuttle screw and a screw nut (Fig. 3a, b). The carrier assembly is thereafter placed in the drive body (generating the drive assembly), and the EIB holding an 18-pin Omnetics connector is subsequently connected to the drive assembly with two fixing screws (Fig. 3c, d). The EIB with the Omnetics connector can be reused (see further below) as long as the gold-pin holes in the EIB are sufficiently intact to allow good connection between the EIB and the tetrodes. An anchoring screw added to the bottom of the drive body (Figs. 3d, 4a) is used for stable fixation of the drive to the dental cement surrounding the craniotomy. One to three anchor screws can be added for this purpose. Before loading of guide and shuttle tubes into the drive assembly, the carrier is moved down to the bottom of the carrier holder by turning (counter-clockwise) of the shuttle screw. A bundle of five guide tubes (> 15 mm long), pre-assembled using superglue, is inserted through the tube hole in the drive body (Figs. 3c, 4a), and through the tube hole in the carrier (Figs. 3c, 4b), leaving < 2 mm of the guide tubes protruding from the top of the carrier (Fig. 4b). The guide tube bundle is glued (superglue) both to the carrier and the drive body (Fig. 4b), and to allow vertical movement of the carrier and the tetrodes, the guide tube bundle is cut into two parts at the bottom of the carrier using fine surgical scissors (Fig. 4c). Individually, four shuttle tubes (> 10 mm long) are inserted into four of the guide tubes (Fig. 4d), leaving < 1 mm of each shuttle tube protruding over the guide tubes. The four shuttle tubes are thereafter carefully glued (superglue) to the top of the guide tubes, avoiding sealing of the shuttle tube openings (Fig. 4d). The guide tubes with the shuttle tubes protruding from the bottom of the drive body are cut to the desired length (suggestively to a protrusion length of 2–3 mm).

One or two ground wires are inserted through the drive body and connected to the EIB with gold pins (Fig. 4e, f). The pre-fabricated tetrodes^13^ are carefully loaded into the shuttle tubes one by one, connected to the EIB using gold pins, and glued to the top of the shuttle tubes (Fig. 4e, f). For the inclusion of optogenetics, an optical fiber coupled to a fiber ferrule^14^ is inserted through the fifth guide tube (Fig. 4g). The optical fiber should protrude from the bottom of the guide tube, and its length be adjusted based on the experimental settings (target brain region, opsin gene, light power density, etc.). The fiber ferrule is secured to the EIB using epoxy (Fig. 4h). The generated tetrode bundle (distance of individual tetrode tips: 0.4–0.5 mm center-to-center) is cut to the desired length (Fig. 4h). To avoid photoelectric artifacts, the uninsulated tips of the tetrodes should be located below the tip of the optical fiber during the electrophysiological recordings in conjunction with optogenetics. For protection of the drive, the 3D printed case is placed over the assembled drive and secured using two anchor screws (Fig. 4i). As a final step, the tips of the tetrodes are gold-plated to lower their impedance^13^. For safe storage and/or transportation, the finished drive can be placed in the 3D-printed container (Fig. 2a). The drive is placed upside down in the bottom container, secured with two anchor screws (Fig. 4j) and the top container is placed over the drive.

### Chronic neuronal recordings in mice and rats

**Figure 5:**
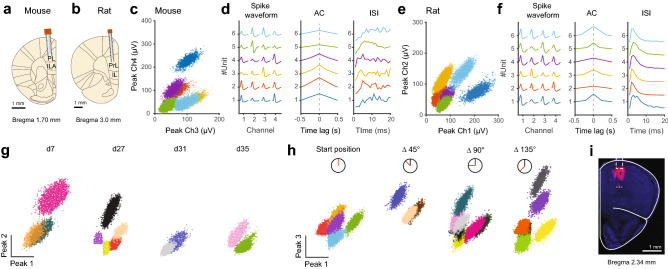
Chronic extracellular single-unit recordings in freely moving mice and rats. (**a**, **b**) Schematic illustration of a coronal section with tetrodes and an optical fiber targeted to the mPFC in a PV-Cre mouse and PV-Cre rat, respectively. (**c**) Peak amplitude plot of action potentials recorded from channel 3 and 4 of a tetrode implanted in a mouse, with color-coding of the identified clusters. (**d**) Average spike waveforms of the single-unit clusters in (**c**), as recorded on the four electrodes of the tetrode. Autocorrelation coefficient (AC) and inter-spike interval (ISI) confirm that the spike times of each cluster belong to individual neurons. (**e**) Peak amplitude plot of action potentials recorded from channel 1 and 2 of a tetrode implanted in a rat, with color-coding of the identified clusters. (**f**) Average spike waveforms of the single-unit clusters in (**e**), as recorded on the four electrodes of the tetrode. AC and ISI confirm that the spike times of each cluster belong to individual neurons. (**g**–**h**) Single-unit recordings in the mPFC of freely moving rats. (**g**) Example recordings from a tetrode discriminating single units 35 days post-implantation. Recordings were performed at 19 different days. The tetrodes were lowered by a 45° turn of the shuttle screw after each recording session. Peak amplitude plots of action potentials recorded from channel 1 and 2 of the same tetrode in four different recording days are shown, with color-coding of the identified clusters. (**h**) Example recordings from a tetrode that was lowered in steps (5 min apart) by 45° turns of the shuttle screw (45° turn: ~ 44 mm). At each depth (n = 4) neuronal signals were recorded, and spikes were clustered (5, 3, 7, and 6 well-isolated single units, respectively) and plotted by the peak amplitude as recorded on electrode 1 and 3 of the same tetrode. (**i**) Tetrode mapping with CM-Dil (red) and DAPI (blue) in the mouse mPFC. Dashed white box: location of implanted tetrodes and an optical fiber. Yellow dashed line: the ventral limitation of CM-Dil labelling, corresponding to the end location of the tetrode tips after tetrode lowering by three full turns of the shuttle screw.

To evaluate the performance of the DMCdrive for neurophysiological recordings in conjunction with optogenetics in freely behaving animals, adeno-associated viral (AAV) vectors with Cre-dependent expression of channelrhodopsin-2 (ChR2) were targeted to the left (1 mouse, 2 rats) or right (1 mouse, 1 rat) medial PFC (mPFC) of transgenic PV-Cre mice^15^ and PV-Cre rats (Supplementary Fig. S1 and Methods). 2–3 weeks later the animals were implanted with a DMCdrive at the same location, with the tetrodes targeted to the prelimbic cortex (Fig. 5a, b and Methods). The activity of mPFC neurons was recorded during freely moving behavior in an open field (25 × 25 cm; Supplementary Fig. S2) during several sessions (5–30 min) over the coming weeks. Analysis of spiking data from single tetrodes in both mice and rats revealed well-separated clusters (Fig. 5c–f), and recording of up to 14 units from a tetrode bundle (four tetrodes) in a single recording session. Recordings (followed by lowering of the tetrodes) were performed in up to 19 different days, with successful recording of single units every day, including 35 days after implantation (the longest time point tested; Fig. 5g, n = 3 rats).

Precise placement and stable control of the tetrodes were important priorities in the design of the drive, as was the possibility to record new neurons over time. To confirm that moving of the tetrodes results in reliable detection of new units, four short (5 min) successive recordings sessions were performed over the course of 50 min in one of the implanted rats 29 days post-surgery (Fig. 5h) After every recording the shuttle screw was turned counter-clockwise 1/8 of a turn (~ 45°), corresponding to lowering of the tetrodes ~ 44 μm. With the use of a manipulator (hex screwdriver) the tetrodes can be moved vertically without risk of damaging of the tetrodes. Further, compared to existing micro-drives holding multiple independently movable tetrodes^7,16^, our single screw-based drive system limits the time used for tetrode movement, reducing the stress put on the animal. Every turn of the screw rendered a distinct electrophysiological condition around the tetrode tip comparable to the conditions of the previous recording (before the turn), with a different number and characteristics of the spiking in each recording, strongly indicative of new neurons being recorded in every session (Fig. 5h). The optogenetic manipulations were analyzed separately (see Fig. 7).

To illustrate the reliability of the tetrode movement in awake animals, we performed an in vivo experiment in which the tips of the tetrodes and the optical fiber were dipped in a red fluorescent dye (CM-Dil) before implantation, for visualization of the tetrode track (n = 2 mice). Using the optical fiber tip as a reference point, the shuttle screw was turned one full turn three times (with appropriate time intervals between every turn), corresponding to the movement of the tetrodes 3 × 350 μm = 1,050 μm (Fig. 5i). Processing of the tissue revealed DiI labelling along the tetrode track all the way down to the final tetrode position. Together these experiments confirm efficient control of the tetrodes and long-term recordings using the DMCdrive.

### Stable single-unit recordings

**Figure 6:**
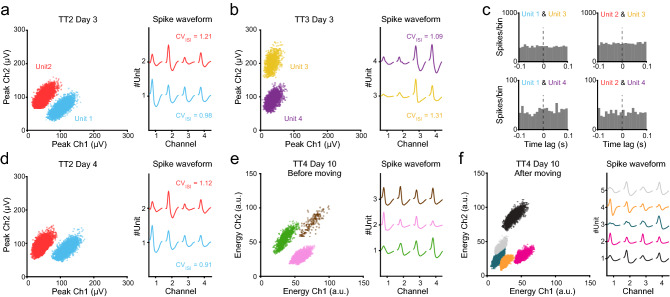
Stable single-unit recordings in freely moving mice and rats. **(a**–**f)** Extracellular recordings of mPFC neurons in two freely moving mice (**a**–**d**: mouse #1, and **e**–**f**: mouse #2). Mouse #1 was implanted with a DMCdrive holding reused EIB parts. **(a)** Left: Peak amplitude plot of spikes recorded from channel 1 and 2 of tetrode 2 (TT2) on the first session of recording (Day 3), with color-coding of two identified clusters. Right: Average spike waveforms of the two single-unit clusters, as recorded on the four electrodes of TT2. **(b)** Left: Peak amplitude plot of spikes recorded from channel 1 and 2 of tetrode 3 (TT3) on the first session of recording (Day 3), with color-coding of two identified clusters. The spikes were recorded simultaneously as the spikes in (**a**). Right: Average spike waveforms of the two single-unit clusters, as recorded on the four electrodes of TT3. **(c)** Cross-correlograms of the spike times of pairs of the units in (**a**) vs (**b**) confirming recording of disparate neurons on the two tetrodes. **(d)** Left: Peak amplitude plot of spikes recorded from channel 1 and 2 of TT2 on the second session of recording (Day 4), with color-coding of two identified clusters. The tetrodes were kept in the same location Day 3 and Day 4. Right: Average spike waveforms of the two single-unit clusters, as recorded on the four electrodes of TT2. **(e)** Left: Energy plot of spikes as recorded on two channels (Ch1 and Ch2) on tetrode 4 (TT4) on Day 10, with color-coding of three identified clusters. The tetrodes were not moved between the implantation of the drive 10 days earlier and the first recording Day 10. Right: Average spike waveforms of the three single-unit clusters, as recorded on the four electrodes of TT4. **(f)** After the recording session in (**e**), the tetrodes were lowered 350 µm, followed by 10 min additional recordings. Left: Energy plot of spikes as recorded on the same channels on tetrode 4 (TT4) as in (**e**), with color-coding of five new clusters. Right: Average spike waveforms of the five single-unit clusters, as recorded on the two channels of TT4 after moving of the tetrodes.

For certain research questions, it can be desirable to record the same neurons across recording sessions. To evaluate the recording stability of the DMCdrive we implanted two mice, as before with the tetrode tips targeted to the prelimbic cortex. To evaluate the feasibility of lowering the experimental costs by reuse of the EIB parts (EIB + Omnetics connector), one of the implanted drives was equipped with EIB parts removed from a used DMCdrive. The optical fiber was omitted in both drives. Three days after implantation the activity of neurons in the prelimbic cortex was recorded (Day 3, 10 min, without preceding movement of the tetrodes) during freely moving behavior in an open field. The recordings were repeated over the next two days (Day 4–5, one session/day), without movement of the tetrodes (Fig. 6a–d). An average of 15 (mouse #1) and 16 (mouse #2) units/session were recorded from the tetrode bundle (four tetrodes), including stable responses of units recorded on the same tetrode on two consecutive days as revealed by analyses of the spike shape features and the coefficient of the variation of the inter-spike-interval (CV_ISI_) (Fig. 6a, d). Cross-correlation analysis of pairs of simultaneously recorded units verified independent recording of disparate neurons on the different tetrodes (Fig. 6a–c).

Reliable electrode repositioning optimizes the unit yield during a recording session, and recovery of new units after long-term retention of the electrode position. Further, recording of a second, deeper brain region, in the same animal can be desirable. To validate the suitability of using the DMCdrive in this type of approaches, we paused the recordings after the third session (Day 5) and resumed the recordings five days later (Day 10). Without preceding movement of the tetrodes, activities in the prelimbic cortex were recorded as before. The tetrodes were thereafter lowered 350 µm by a 360° turn (counter-clockwise) of the shuttle screw, followed by a second 10 min recording. Unit activity was recovered in both recordings, with different units being recorded in the two sessions (Fig. 6e, f). Together, these experiments demonstrate that the DMCdrive provides good recovery of single-unit activity over several consecutive days, and allows across-days recording of neurons. Units can be recorded without repositioning of the tetrodes, including after days with static tetrode positioning, and movement of the tetrodes allows recording of new units. Further, the drive design ensures the recording of disparate neurons on the different tetrodes. In addition, the EIB parts can efficiently be reused without affecting the unit yield, contributing to reduced experimental costs. In the recording experiments described, a total of 239 units were recorded from mice (n = 4 mice, 17 recordings sessions in total, 3–4 sessions/mouse), and 75 units from rats (n = 3 rats, 10 recording sessions in total, 1–5 sessions/rat). The units were classified based on spike waveform features, revealing recording of both narrow-spiking, putative inhibitory interneurons, and wide-spiking, putative excitatory mPFC units (Supplementary Fig. S3).

### Optogenetic modulation of single-unit and LFP activity

**Figure 7:**
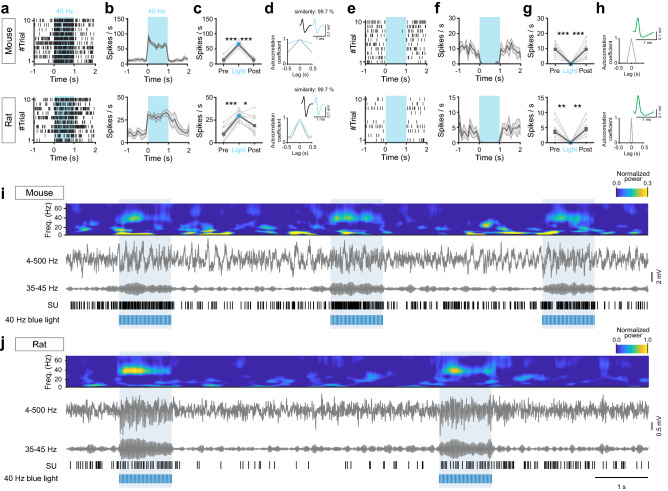
Optogenetics in conjunction with chronic single-unit and LFP recordings. (**a**–**h**) Optogenetic manipulation of ChR2-expressing mPFC PV interneurons in PV-Cre mice and PV-Cre rats. Top: mouse, bottom: rat. (**a**–**d**) Light-activated mPFC PV interneurons. (**a**) Spike raster of an example light-responsive PV interneuron responding to 40 Hz blue light (473 nm, 1 s, 3 ms pulses) application with significantly increased spiking. (**b, c**) Average modulation of the neuron in (**a**) across 10 trials. (**d**) Temporal structure of the spiking of the neuron in (**a**–**c**). Inset: The average spike waveform of the recorded neuron. Spontaneous (gray) and light-evoked (blue) action potential waveforms exhibit very high similarity. (**e**–**h**) Concurrently recorded WS putative excitatory neurons in the local mPFC circuitry. (**e**) Spike raster of an example WS neuron demonstrating significantly decreased spiking in response to light-activation of local PV interneurons. (**f, g**) Average modulation of the neuron in (**e**) across 10 trials. (**h**) Temporal structure of the spiking of the neuron in (**e**–**g**). Inset: The average spike waveform of the recorded neuron. (**i**, **j**) Optogenetic manipulation of ChR2-expressing mPFC PV interneurons. Spectrogram (4–70 Hz), raw LFP (4–500 Hz), band-pass filtered LFP (35–45 Hz), and single-unit (SU) activity from mPFC recordings (10 s) in freely moving PV-Cre mice (**i**) and PV-Cre rats (**j**). Blue light application (473 nm, 1 s, 40 Hz, 3 ms pulses) results in increased oscillatory activity in the gamma range as seen in the LFP. Same SUs as in (**a**). Shaded area and error bars: ± SEM. *p < 0.05, **p < 0.01, ***p < 0.001 by paired t-test.

Cortical PV interneurons are important circuit regulators, and experiments employing optogenetics in intact mice have been central to demonstrate the role of this neuronal population in the generation of gamma oscillations, circuit functions, cognition and behavior^12,15,17,18^. Here we combined recordings with opto-tagging and optogenetic drive of PV interneurons in the mPFC in mice and rats using the DMCdrive. Application of 40 Hz blue light (473 nm, 3 ms light pulse, 1 s duration, 5–10 mW) resulted in a significantly increased short-latency firing in a set of neurons in both rats and mice (Fig. 7a–d). The light-evoked action potentials were characteristic of prefrontal fast-spiking (FS) PV interneurons, with short half-trough width and comparable peak and trough deflections^15^ (Fig. 7d). Comparison of spontaneous and light-activated action potentials revealed very high similarity (99.7%) between the spike waveforms^19^, demonstrating true light-activation of ChR2-expressing PV interneurons (Fig. 7d). Prefrontal inhibitory PV interneurons provide strong inhibition onto excitatory neurons in the local network^15^, and in agreement with this application of blue light resulted in silencing of nearby neurons with waveform properties typical of cortical excitatory neurons^12,20^ in both mice and rats (Fig. 7e–h).

The LFP activity provides information about the activity of aggregates of neurons, including the temporal scales of the activities^21^. It has been firmly established that synchronous activity of cortical PV interneurons generates gamma oscillations (30–80 Hz) in the local network^22,23^. Optogenetic studies have shown that activation of PV interneurons results in an increase of the LFP power specifically at gamma frequencies^12^. We here activated prefrontal PV interneurons with blue light at a typical gamma frequency (40 Hz, 3 ms light pulse, 1 s duration, 5–10 mW) in freely moving mice and rats. Analysis of the LFP revealed increased LFP power and gamma activity time-locked to the light application (Fig. 7i, j). In agreement with the mechanisms of fast-spiking PV interneurons generating cortical gamma rhythms, light-activated neurons with increased firing displayed typical narrow-spiking (NS) features (Fig. 7d).

## Discussion

Electrophysiological recordings in combination with local optogenetic manipulations in freely behaving animals allow investigation of neuronal mechanisms underlying brain functions, and have been used to probe emotion, memory, attention, and perception, and more^15,18,24,25^. The ability to tag neurons with light for identification of activity patterns of select neuronal populations has greatly aided the understanding of how specific neuron types contribute to behavior^26,27^. Tetrode arrays have long been the gold standard for in vivo neuronal recordings and provide high temporal (sub-millisecond) and spatial (single-unit) resolution. Micro-drive systems with movable tetrodes enable high-yield and long-term recordings over the course of days to weeks, and the integration of fiber optics allows control of neurons or neural circuits during recordings. Application in freely behaving rodents puts constraints on the size and weight on the implant, and as a general rule an implant can only be used in a single animal, even though some parts can be reused. Implants, thus, must regularly be fabricated or purchased.

We here present an affordable simple-to-operate, truly easy-to-build, compact, and low-weighted micro-drive for reliable multi-tetrode electrophysiological recordings in combination with optogenetic investigations. The DMCdrive consists primarily of 3D-printed components, remarkably reducing the cost of the device. The 3D-printing in addition provides possibilities for desired customizations. The 3D-printed drive parts are configured for very straightforward assembly and reduced drive build time. To minimize friction between the tetrodes and the guide tubes, and to provide predictable linear motion of the tetrodes, the tetrodes are disposed vertically and parallel to each other, with all tetrodes being moved together by turning of a shuttle screw. In contrast to other micro-drives intended for tetrode recordings^7,28,29^, the connections of the electrodes to the EIB in the DMCdrive are protected from the external environment by a 3D-printed case. Further, the use of a tetrode carrier and a single screw-based system for tetrode movement avoids the need to use tools (e.g., a screwdriver) in close vicinity of the electrodes for tetrode movement, limiting the risk of irreversible damaging the electrodes. As other single screw-based drive designs^11,30,31^, the DMCdrive entails low fabrication costs and easy operation. Additional strongpoints of the DMCdrive are the reliability and stability of the neurophysiological recordings and optogenetic manipulations, and the particular easy and fast drive building, making the drive design a genuinely competitive alternative.

The presented design is optimized for chronic recordings in freely moving mice and includes only four tetrodes, but the drive design can flexibly be modified to accommodate up to eight tetrodes suitable for multi-site recordings, or larger scale recordings in rats. As demonstrated, the original design can nevertheless be proficiently used in rats, although rats would be able to carry larger designs holding more tetrodes. The size and weight of the drive have been reduced to a minimum, limiting the effects on animals’ behavior, and the rectangular shape of the implant is particularly convenient for lowering of tetrodes and connecting/disconnecting cables in awake animals. The main possible limitation of the drive design is the permanent fixation of the fiber optic to the device, prohibiting movement of the optical fiber. Integration of movable optical fibers into the recording array, while avoiding increases in the size, weight, and complexity of the drive, yet imposes a great technical challenge for in vivo electrophysiologists. As the choice of opsin gene, the functional expression levels of the opsin, and the light power density reaching the light-sensitive neurons all are factors influencing the efficacy of light-based control of neuronal activity, the parameters in individual experiments will determine how far from the optical fiber the tetrode tips can be lowered with preserved optical manipulations^32^. The tetrodes in the DMCdrive design can be lowered up to 4 mm and the recording sites can, thus, be lowered beyond the range of light-induced activation of e.g., ChR2 expressing neurons^33^. Fortunately, novel opsins with increased light-sensitivity are regularly being developed, and recent additions can induce spiking activity > 3 mm from the optical fiber tip^34^, reducing both the need for, and benefits of, movable optical fibers.

To date, various off-the-shelf and in-house-built devices to support chronic multiple tetrode recordings together with optical stimulation have been designed, including the VersaDrive (Neuralynx), systemDrive, SLIQ hyperdrive, flexDrive, and more^7,28,31,35,36^. Compared to the DMCdrive, these devices can carry a larger number of recording channels and allow positioning of each tetrode independently. However, as the number of tetrodes increases, so does the cost and the complexity of the drive. Further, the drive complexity greatly affects the time and effort needed for tetrode/fiber optic loading and building of the device. In addition, the bending of the tetrodes and the concentric arrangement of tetrode bundles in some devices^7,28,29^ are more likely to impose friction between a tetrode and the guide tube during tetrode lowering, negatively impinging on the reliability of the tetrode movement. Our design does not support individually movable tetrodes, which is often the method of choice for high-yield recording of neurons from multiple brain structures and layers (e.g., hippocampal formation). On the other hand, the DMCdrive with its relatively small size and low drive body weight can avoid the need of a counterweight pulley system (no counterweight pulley system was used in the presented experiments), and provides highly precise targeting of brain regions without tetrode bending.

The observation of new neurons by repositioning of the electrodes after the potential formation of a glial sheath shielding the electrodes from active neurons^5,6^ (peaking around day seven post implantaion^37^) is rarely reported for micro-drive designs. We here show that the DMCdrive allows for successful recording of new neurons, upon repositioning of the tetrodes, after 10 days of retention of the tetrode positions. Given the outlined, and highly intentional, advantages the DMCdrive offers, we firmly believe that the described drive is a welcomed addition to the toolbox in experimental neuroscience and will find many application areas, particularly for users with limited prior experience of neurophysiology in behaving rodents.

## Methods

### Animals

All procedures were approved and performed in accordance and compliance with the guidelines of the Stockholm Municipal Committee for animal experiments. Adult PV-Cre mice (males, 3–4 months old; Jax Stock no. 008069; https://jax.org/strain/008069) and PV-Cre knock-in rats (manuscript in preparation; male and female, 2–4 months old) were used. Animals were housed under a standard 12-h light/dark cycle.

### General surgical procedures and viral injections

For all surgeries (viral injection and drive implantation), the animals were injected with buprenorphine (0.05 mg/kg s.c.) 30 min prior to surgery. Animals were anesthetized with ~ 2% Isoflurane in oxygen, fixed in a stereotaxic device, and lidocaine (maximum 5 mg/kg) was injected locally before skin incision. A craniotomy (1.0–1.5 mm diameter) was unilaterally made above the mPFC (mice: 1.7 mm anterior to Bregma and 0.25 mm lateral to midline, rats: 3.0 mm anterior and 0.5 mm lateral to the midline). For optogenetic activation of PV-expressing interneurons an adeno-associated viral vector with Cre-dependent expression of ChR2, AAV-DIO-ChR2-mCherry (rAAV5/EF1a-DIO-hChR2(H143R)-mcherry; 4 × 10e12 viral particles/ml, UNC Vector Core) was injected (0.5 µl) into the mPFC, centered to the prelimbic cortex, (mice: AP 1.7 mm, ML 0.25 mm, DV − 1.0 mm; rats: AP 3.0 mm, ML 0.5 mm, DV − 2.0 mm) using a glass micropipette coupled to a motorized stereotaxic injector (Stoelting) at a rate of 0.1 µl/min. The glass pipette was kept in place for 5 min after injection and then slowly withdrawn to minimize backflow. The skin was closed with tissue glue (Vetbond, 3 M) or sutures (silk, Ethicon). Carprofen (5 mg/kg) was administered subcutaneously to the animals before recovery from anesthesia, and 24 h after surgery.

### Drive implantation

For mice and rats included in optogenetic experiments, a DMCdrive was implanted three weeks after viral injections, following the general surgical procedures (see above). The implant was positioned in the craniotomy made prior to the virus injection. For animals (mice, n = 2) not subjected to optogenetic manipulations, the craniotomy and the drive implantation were conducted during a single surgery. The craniotomy was covered with vaseline to protect the tetrodes and the optical fiber. Stainless steel skull screws (3 for mice and 5 for rats; Supplementary Fig. S4) and dental cement were used for firm attachment of the drive to the skull. The ground wire (uncovered part) of the drive was wrapped around the skull screws as ground. To protect the EIB and the exposed parts of the drive from physical contact with the environment, the top of the implant (the opening of the case) was sealed with clear office tape. The tape provides easy unsealing/resealing of the micro-drive and avoids the need for e.g., a plastic lid fixed with screws. To exclude the risk of other animals damaging the implant, the animals were single-housed in individually ventilated cages (IVC) after the implant surgery.

### Tissue processing

For perfusions, the animals were deeply anesthetized with pentobarbital and transcardially perfused with 0.1 M phosphate buffer (PB) followed by 4% paraformaldehyde (PFA) in 0.1 M PB. For analysis of Cre-recombination in transgenic PV-Cre rats, 50 mm thick brain sections were cut on a vibratome (Leica VT1000, Leica Microsystems). The cut tissue was permeabilized with 1 × TBST (0.3% Triton-X in 1 × TBS) for 1 h, blocked with 10% normal donkey serum in 1 × TBST for 1 h, and thereafter incubated with primary antibodies (1:1,000 Parvalbumin, guinea pig, Swant, code no. GP72, 1:1,000 RFP, rabbit, Rockland, code no. 600-401-379) in 1 × TBST at room temperature for 12–24 h. The sections were thereafter washed three times in 1 × TBST and incubated with a species-specific fluorophore conjugated secondary antibody (1:500 donkey anti-guniea pig, DyLight 488, Jackson, and 1:500 donkey anti-rabbit, Cy3, Jackson) in 1 × TBST for 3–5 h. The sections were thereafter consecutively washed with 1 × TBST, 1 × TBS and 1 × PBS (10 min each). Vibratome cut sections were mounted on glass slides (Superfrost Plus, Thermo Scientific). All sections were coverslipped (Thermo Scientific) using 50:50 glycerol:1 × PBS.

### Determining tetrode placement

To illustrate the achieved control and precision of depth movement of the tetrodes in freely moving animals, a DMCdrive was implanted in the dorsal PFC of a mouse. Prior to the implantation, the tetrodes tips were aligned with the optical fiber, and carefully dipped in CellTracker™ CM-Dil fluorescent dye (ThermoFisher). 24 h post-surgery, the tetrodes were moved down ~ 1 mm by three full (360°) turns of the shuttle screw (with appropriate time intervals between every turn, corresponding to movement of the tetrodes 3 × 350 μm = 1,050 μm). One hour later the mouse was transcardially perfused. 300 μm thick brain sections were cut on a vibratome (Leica VT1000, Leica Microsystems). Brain sections were cleared according to a modified CUBIC clearing method^38^. Z-stack images were acquired at 10x, using a Zeiss (LSM800) confocal laser scanning microscope.

### Neural data acquisition and optogenetic manipulation

Single-unit and LFP activities were recorded using a Cheetah data acquisition system (Digital Lynx 4SX, Neuralynx). Unit signals were amplified (× 10,000), band-pass filtered (600–6,000 Hz), digitized (32 kHz), and stored on a PC for off-line analysis. LFP signals were acquired from one channel of each tetrode at 32 kHz sampling frequency, and band-pass filtered between 0.1 and 500 Hz. For optogenetic experiments, blue light (473 nm, 3 ms pulse width, 1 s duration, 5–10 mW at the fiber tip, 40 Hz) was delivered through the optical fiber using a DPSS laser (Cobolt).

### Spike sorting

To identify single units from tetrode data, spikes were manually sorted based on various spike waveform features (peak amplitude, total energy, principal components, etc.) using MClust (offline spike sorting software, written by A. D. Redish). Only well-separated single units defined by isolation distance > 15, L-ratio < 0.2, and the spikes < 0.01% with < 2 ms^39^ were used in the data analysis.

### Data analysis

All data analysis was performed using custom scripts written in MATLAB (MathWorks). To assess the effects of optogenetic stimulation on the activation of mPFC neurons, neuronal activity relative to the light events was expressed in a peri-event time histogram (PETH) and a spike raster (rastergram) for each neuron. To examine light artifact in light-excited units, the similarity of spike waveform shapes for 1 s before and after the light onset was determined by calculating the correlation coefficient (*r*) between two average spike waveforms for each unit. For spectral analysis, LFP signals were down-sampled to 1 kHz. The LFP spectrogram (4–100 Hz) was computed by convolving the LFP signal with a complex Morlet wavelet. All values are given as mean ± SEM unless otherwise stated.

## Supplementary information

Supplementary file1 (PDF 9166 kb)

## Data Availability

The datasets generated and/or analyzed in the current study are available from the corresponding authors upon reasonable request.
