# Supplementary material for: The DMCdrive: practical 3D-printable micro-drive system for reliable chronic multi-tetrode recording and optogenetic application in freely behaving rodents
Source: Sci Rep. 2020 Jul 16;10:11838. doi: 10.1038/s41598-020-68783-9 (PMC7366717; doi:10.1038/s41598-020-68783-9)
Supplement: Supplementary file 1 — Supplementary file1 (PDF 9166 kb) [file 41598_2020_68783_MOESM1_ESM.pdf]

Supplementary information

The DMCdrive: practical 3D-printable micro-drive system for reliable chronic multi-tetrode recording and optogenetic application in freely behaving rodents

Hoseok Kim[1,3*], Hans Sperup Brünner[1,3] & Marie Carlén[1,2*]

[1]Department of Neuroscience, Karolinska Institutet, 171 65 Stockholm, Sweden
[2]Department of Biosciences and Nutrition, Karolinska Institutet, 141 83 Huddinge, Sweden
[3]These authors contributed equally to this work.
*Correspondence: kim.hoseok@ki.se, marie.carlen@ki.se

Supplementary Figure S1.

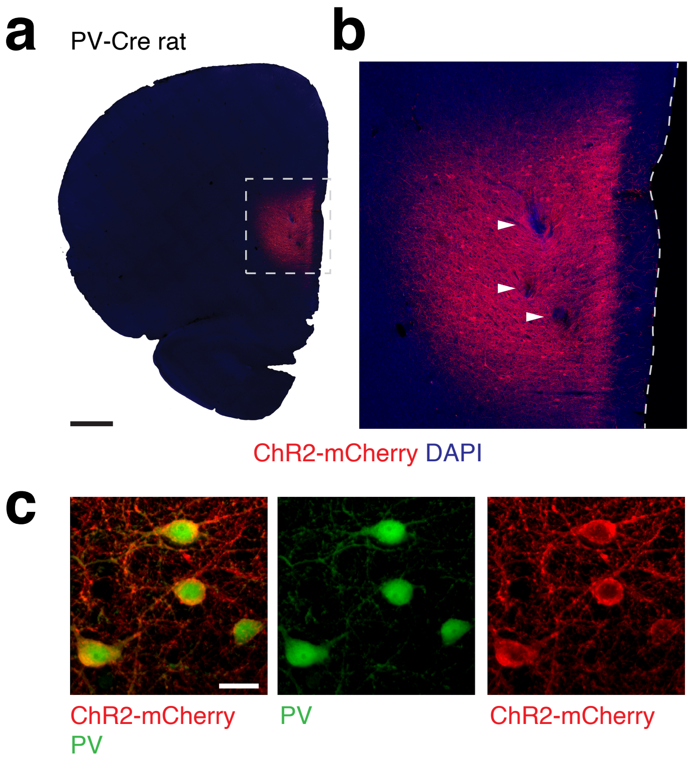

Supplementary Figure S1. Transgenic PV-Cre knock-in rats

A transgenic knock-in rat line with Cre recombinase targeted to neurons expressing the calcium binding protein parvalbumin (PV) was generated using CRISPR technology (manuscript in preparation). Direct investigation of Cre recombination in PV neurons by injection of an adeno-associated virus (AVV) with Cre-dependent expression of ChR2-mCherry into the mPFC reveal robust and specific expression of ChR2 in mPFC PV interneurons (97.88 ± 0.54% of the ChR2-mCherry neurons express PV).

(**a**) Representative image of PV-Cre rat injected with AAV-DIO-ChR2-mCherry targeted to the mPFC and implanted with a DMCdrive at the same location for integration of electrophysiological recordings with optogenetic manipulations during freely moving behavior.

(**b**) Magnification of the outlined area in (**a**). Arrowheads; track marks from the tetrodes.

(**c**) Representative images of ChR2-mCherry (red) expressing mPFC PV interneurons (green) after immunohistochemistry (see **Methods**).

Scale bars: 500 μm in (**a**) and 20 μm in (**c**).

Supplementary Figure S2.

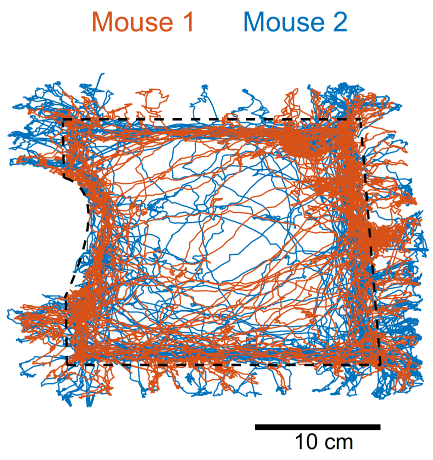

Supplementary Figure S2. Tracking of movement in the open field

Example tracking of the movement (15 min) in an open field (25 x 25 cm, 5 cm wall height) of two mice implanted with a DMCdrive. A point between the animals' ears was tracked using DeepLabCut[1]. Black dashed line: outline of the open field. The traces reveal that the animals extend the head over the low walls of the track and extensively explore the environment outside of the track.

Reference

1. Mathis, A. *et al.* DeepLabCut: markerless pose estimation of user-defined body parts with deep learning. *Nat. Neurosci.* 21, 1281–1289 (2018).

Supplementary Figure S3.

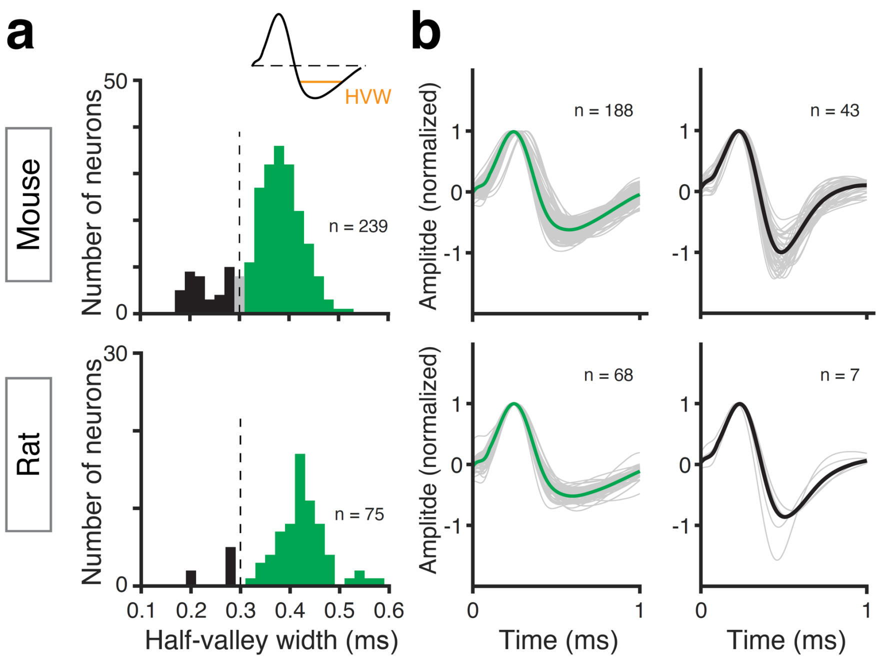

Supplementary Figure S3. Spike waveform-based classification of all mPFC neurons recorded in freely moving mice and rats

Top: mice (n = 4), bottom: rats (n = 3).

(**a**) Histograms of the half-valley waveform (HVW) duration of the recorded neurons (mice: n = 239, rats: n = 75). The neurons were classified based on the HVW of spike waveforms, identifying narrow-spiking (NS, < 0.29 ms) putative inhibitory interneurons (black) and wide-spiking (WS, > 0.31 ms) putative excitatory neurons (green). Dashed line: 0.3 ms, gray: non-classified neurons.

(**b**) Average spike waveforms of the classified WS (green) and NS neurons (black). Light gray: individual neurons.

Supplementary Figure S4.

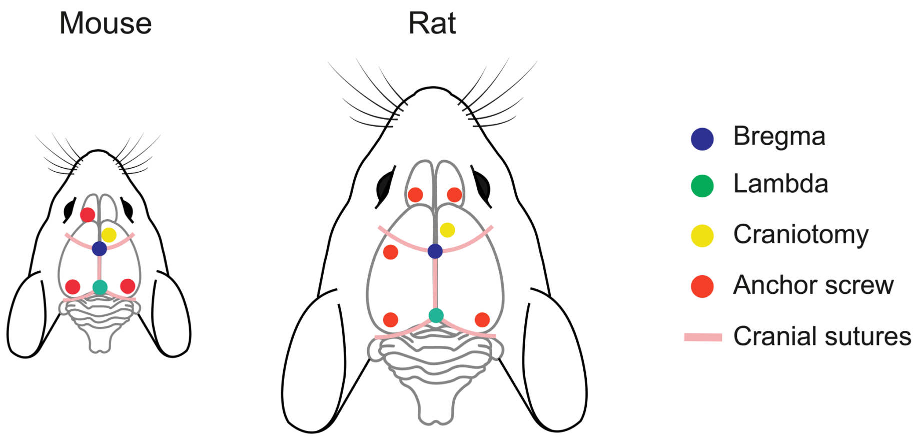

Supplementary Figure S4. Positioning of anchor screws

Illustration of anatomical landmarks and the positioning of anchor screws for implantation of a DMCdrive and recordings in the mPFC of rats and mice, respectively.
